# Factors Associated with Persistent Metatarsal Pain in Patients with Rheumatoid Arthritis in Remission: Clinical Implications and Multivariable Analysis in a Cross-Sectional Study

**DOI:** 10.3390/biomedicines13082024

**Published:** 2025-08-20

**Authors:** Rebeca Bueno Fermoso, Maria Rosario Morales Lozano, Carmen Martínez Rincón, Pablo García-Fernández, Juan Miguel López González, Maria Luz González Fernandez

**Affiliations:** 1 Faculty of Nursing, Physiotherapy and Podiatry, Complutense University of Madrid, 28040 Madrid, Spain; rebueno@ucm.es (R.B.F.); rmorales@ucm.es (M.R.M.L.); nutrias@enf.ucm.es (C.M.R.); luzalez@ucm.es (M.L.G.F.); 2 Hospital Universitario General de Villalba, 28400 Collado Villalba, Spain; jmlg2097@gmail.com

**Keywords:** rheumatoid arthritis, metatarsalgia, foot pain, remission, joint damage, synovitis

## Abstract

**Background**: Foot pain often persists in patients with rheumatoid arthritis (RA), even during clinical remission. However, its causes are not fully understood. Identifying factors specifically associated with metatarsal pain, rather than generalized foot pain, may improve targeted management strategies. **Objectives**: The aim of this study was to compare the clinical, biomechanical, and radiological characteristics of RA patients in remission with isolated metatarsal pain versus those with pain in other foot regions, and to identify independent predictors of metatarsal pain. **Methods**: This cross-sectional study included 118 RA patients in remission, classified into two groups: isolated metatarsal pain (*n* = 61) and pain in other foot regions (*n* = 57). Clinical variables (demographics, disease duration, treatment, comorbidities), biomechanical measures (ankle, first metatarsophalangeal and subtalar joint mobility, hallux valgus severity, foot type), radiographic findings (erosions, subluxations), and ultrasound-detected synovitis in the 2nd–5th metatarsophalangeal (MTP) joints were recorded. Independent predictors were identified using binary logistic regression. **Results**: Patients with metatarsal pain had higher rates of severe hallux valgus, MTP synovitis, and dislocations ≥ 50%. Independent predictors were hallux valgus (OR = 5.428, 95% CI: 1.528–19.287, *p* = 0.009), MTP synovitis (OR = 2.093, 95% CI: 1.337–3.275, *p* = 0.001), and MTP dislocations (OR = 2.092, 95% CI: 1.275–3.432, *p* = 0.003). **Conclusions**: Persistent metatarsal pain in RA remission is associated with a distinct structural and biomechanical profile. Comparing foot pain by location may help identify clinically relevant patterns and support more individualized assessment and treatment strategies. Due to the cross-sectional design, causality cannot be established.

## 1. Introduction

Rheumatoid arthritis (RA) is a chronic, systemic autoimmune disease primarily affecting synovial joints, leading to structural damage, functional decline, and disability [1]. In 2019, over 18 million people worldwide lived with RA [2], a figure expected to rise significantly by 2050 [1].

Foot involvement occurs in up to 90% of RA patients during the disease course [3,4,5,6], often as the initial symptom [7,8], and contributes substantially to long-term disability [9].

Although pharmacological advances aim for clinical remission and joint protection, many patients still report foot pain and show signs of subclinical synovitis [10,11,12,13,14,15]. This mismatch may be due to biomechanical dysfunction and residual structural damage, particularly in weight-bearing joints [16,17,18,19].

Foot involvement in RA is heterogeneous. The forefoot is commonly affected, but clinical manifestations vary [20,21,22,23,24,25]. As Matsumoto et al. [26] suggested, structural and biomechanical characteristics may differ by location, though this remains understudied.

Understanding biomechanical and structural contributors to forefoot pain in RA remission may support earlier, more targeted interventions. It also remains unclear whether specific patterns of articular damage are directly related to pain localization or whether distinct features explain why metatarsal pain persists in otherwise well-controlled disease. This study compares clinical, biomechanical, and radiographic features in RA patients in remission with isolated metatarsal pain versus other foot regions and identifies independent predictors of metatarsal pain.

## 2. Materials and Methods

### 2.1. Study Design and Participants

#### 2.1.1. Study Design

A case–control study was conducted following the Declaration of Helsinki and Good Clinical Practice guidelines. Ethical approval was granted by the Clinical Research Ethics Committee of Hospital Clínico San Carlos, Madrid, Spain (protocol 21/719-E) on 21 December 2021. All participants provided written informed consent prior to inclusion.

#### 2.1.2. Context

The study was conducted between June 2023 and December 2024 at the Rheumatic Foot Unit of the University Podiatry Clinic, Faculty of Nursing, Physiotherapy and Podiatry, Complutense University of Madrid.

#### 2.1.3. Participants

The study population consisted of patients diagnosed with rheumatoid arthritis (RA) by their rheumatologist according to the 2010 ACR/EULAR classification criteria [18]; the patients were referred from five public hospitals. All participants reported foot pain and were in clinical remission based on the Disease Activity Score-28 (DAS28) [27,28] and Clinical Disease Activity Index (CDAI).

Patients with isolated metatarsal pain were assigned to the case group, while those with pain in other foot regions were included as controls. Individuals reporting pain in multiple foot areas, including the metatarsal region, or pain limited to the first metatarsophalangeal joint were excluded.

#### 2.1.4. Study Size

Prior studies reported a markedly higher prevalence of forefoot pain compared to other foot regions in RA patients. Bouysset et al. [20] found 82.5% involvement of the forefoot vs. 21% of the tarsus, and Vidigal et al. [29] reported 70% vs. 32% in the midfoot. Based on these differences (>30% between regions), the sample size calculation indicated that at least 28 patients per group would be required to detect a statistically significant difference (α = 0.05, power = 80%).

Patients were divided into two groups according to pain location: forefoot pain group (*n* = 61) and other foot pain group (*n* = 57). The total sample (*n* = 118) exceeded the minimum requirement. All patients were consecutively recruited from five public hospitals in Madrid, Spain.

#### 2.1.5. Inclusion and Exclusion Criteria

Inclusion criteria: Age ≥ 18 years, RA diagnosis confirmed by a rheumatologist, current foot pain, and recent weight-bearing dorsoplantar radiographs (<12 months).

Exclusion criteria: Other rheumatic/neuromuscular diseases, diabetes, ulcers/tumors, recent trauma or surgery (<6 months), orthotic use or corticosteroid injection (<3 months), cognitive impairment, multifocal foot pain including metatarsals, or inability to complete the assessment.

### 2.2. Data Collection and Procedure

#### 2.2.1. Sample Selection

Investigator 1 screened patients, confirmed eligibility criteria, and obtained written informed consent. Investigator 2 assessed the location of foot pain and selected the foot with the most localized pain, excluding cases with multifocal pain or pain limited to the first metatarsophalangeal joint (MTP1). Radiographs of the selected foot were anonymized. Investigator 3, blinded to clinical data, performed the ultrasound and radiographic evaluations.

A total of 180 patients with a confirmed diagnosis of RA and foot pain were initially evaluated. For each patient, the foot with the most localized pain was selected, provided the pain was confined to a single anatomical region (forefoot, hindfoot, medial midfoot, or lateral midfoot).

Patients were excluded due to pain in multiple foot regions (*n* = 19), absence of recent radiographs (*n* = 9), corticosteroid infiltration within the previous three months (*n* = 8), ongoing orthotic treatment (*n* = 7), exclusionary comorbidities (*n* = 5), prior foot surgery (*n* = 9), or active RA flare at the time of assessment (*n* = 5).

The final sample comprised 118 patients: 61 with isolated metatarsal pain (ARm group) and 57 with pain in other foot regions (ARo group).

#### 2.2.2. Clinical Assessment:

Data on sociodemographics, disease duration, treatment, comorbidities, smoking, alcohol use, and physical activity were collected. Sedentary behavior was classified per WHO guidelines based on age and chronic condition [30].

Pain intensity was measured using a Visual Analog Scale (VAS, 0–10) [31]. Pain was classified as acute (<3 months) or chronic (≥3 months).

#### 2.2.3. Joint Mobility

Ankle dorsiflexion was measured using the Silfverskiöld test [32,33] in supination to avoid compensation. Limitation was defined as <90° (knee extended) or <110° (knee flexed), with both positions considered equivalent in the analysis. Reported intra-rater reliability ranges from 0.708 to 0.828 [32].

First MTP joint mobility was assessed in a non-weight-bearing position with a goniometer [34]. Dorsiflexion ≥ 60° was normal; <40° indicated hallux limitus, and <10° hallux rigidus [35]. Both were classified as mobility limitations.

#### 2.2.4. Digital Deformities

Lesser toe deformities (claw toe, hammer toe, mallet toe, and swan-neck deformities) were recorded [36], along with the presence of a tailor’s bunion (fifth-toe deformity) [37].

Hallux valgus severity was graded using the validated Manchester scale [38].

#### 2.2.5. Ultrasound Scanning

Ultrasound examinations were performed by the same experienced podiatrist, with the patient seated, the knee flexed at 90°, and the foot placed flat on the examination table. Synovitis and power Doppler signal were assessed dorsally at the 1st to 5th metatarsal heads in accordance with EULAR-OMERACT guidelines [39]. The scans were conducted using a Samsung 500 ultrasound system equipped with an 8–12 MHz linear transducer.

#### 2.2.6. Radiological Assessment

Weight-bearing dorsoplantar radiographs were evaluated by the same podiatrist with expertise in rheumatic foot disorders. The presence of erosions was recorded at the 1st to 5th metatarsophalangeal (MTP) joints and the interphalangeal joint of the hallux, based solely on their presence or absence [40]. Subluxations and dislocations of the 2nd to 5th MTP joints were also assessed and categorized as mild (<50% joint displacement), severe (>50% displacement), or complete dislocation [41]. For statistical analysis, severe subluxations and dislocations were grouped together as one category.

### 2.3. Bias

All evaluations were conducted by trained podiatrists with over 10 years of clinical experience in rheumatic foot pathology, ensuring consistency in both clinical and imaging assessments. To minimize intra-rater variability, joint mobility was measured using a standardized goniometer protocol.

Ultrasound and radiographic evaluations were performed by a single examiner and conducted in a blinded manner to reduce observer bias. Inter-observer variability was minimized by centralizing all imaging assessments in one experienced investigator.

### 2.4. Statistical Analysis

Statistical analyses were performed using SPSS version 25.0 (IBM Corp., Armonk, NY, USA). Quantitative variables were expressed as means ± standard deviations (SDs), and categorical variables as frequencies and percentages. The Kolmogorov–Smirnov test was applied to assess the normality of continuous data distributions.

Group comparisons were carried out using the Chi-square test (or Fisher’s exact test, when appropriate) for categorical variables, and either Student’s t-test or the Mann–Whitney U test for continuous variables, depending on the distribution. A two-tailed *p*-value < 0.05 was considered statistically significant.

A binary logistic regression analysis was then conducted to identify clinical, biomechanical, and radiographic variables independently associated with metatarsal pain. Variables that were statistically significant in univariate analyses or deemed clinically relevant were included in the multivariate model. Model fit was evaluated using the Hosmer–Lemeshow test, and the proportion of explained variance was assessed using the Nagelkerke R^2^. Odds ratios (ORs) with 95% confidence intervals (CIs) were reported for all predictors retained in the final model.

Collinearity among independent variables was assessed using Pearson’s correlation coefficients, tolerance values, and variance inflation factor (VIF), considering |r| > 0.70, tolerance < 0.10, or VIF > 10 as indicative of problematic collinearity.

## 3. Results

The final sample included 118 patients: 61 (51.6%) with isolated metatarsal pain (ARm group) and 57 (48.3%) with pain in other foot regions (ARo group). The mean disease duration was comparable between groups: 18.62 ± 14.65 years (ARm) and 18.75 ± 13.65 years (ARo).

In the ARo group, pain was distributed as follows: posterior tibial tendon (*n* = 15, 12.8%), peroneal tendons (*n* = 4, 3.4%), heel (*n* = 12, 10.2%), ankle (*n* = 15, 12.8%), and dorsal tarsus (*n* = 6, 5.1%).

Women represented 93.2% of the total sample: 90.1% in the ARm group and 96.4% in the ARo group. The mean age was similar between groups (62.86 ± 12.59 vs. 62.15 ± 11.51 years, respectively).

Pain was chronic in almost all cases. Bilateral pain was the most common presentation, although unilateral pain was reported by six patients (9.2%) in the ARm group and four patients (7.1%) in the ARo group. Mean pain intensity (VAS) was also similar: 7.1 ± 2.08 (ARm) vs. 6.96 ± 2.2 (ARo).

Anthropometric characteristics showed no significant differences: BMI was 25.87 ± 4.20 kg/m^2^ (ARm) and 26.42 ± 3.70 kg/m^2^ (ARo).

Comorbidities such as hypercholesterolemia (26.1% vs. 39.2%), hypothyroidism (6.1% vs. 19.6%), and hypertension (29.2% vs. 28.5%) were similarly distributed between groups.

Lifestyle factors, including smoking, alcohol intake, and sedentary behavior, were also comparable.

Paracetamol use was significantly more frequent in the ARo group compared to the ARm group (33.3% vs. 13.1%, *p* = 0.009). Likewise, synthetic DMARDs were more commonly used in the ARm group (68.8% vs. 47.4%, *p* = 0.018). No significant differences were observed in the use of biologics, corticosteroids, NSAIDs, or other supplements. Detailed medication and lifestyle factors are provided in Table 1, and detailed clinical, biomechanical, and radiological characteristics are provided in Table 2.

No positive power Doppler signal was detected in any of the evaluated MTP joints.

A binary logistic regression model was conducted to identify variables independently associated with localized metatarsal pain (ARm) in patients with rheumatoid arthritis. The model included demographic, clinical, biomechanical, and radiological predictors.

The final model was statistically significant (χ^2^ = 56.905, df = 13, *p* < 0.001), indicating that it reliably distinguished between the ARm and ARo groups. The Hosmer–Lemeshow test confirmed a good model fit (χ^2^ = 6.308, *p* = 0.613). The model explained 51.0% of the variance (Nagelkerke R^2^) and correctly classified 78.0% of cases.

Collinearity among predictors included in the final model was assessed using Pearson’s correlation coefficients, variance inflation factor (VIF), and tolerance values. The highest correlation was observed between dislocations/subluxations >50% and erosions (r = 0.580; 95% CI: 0.446–0.689), below the commonly accepted threshold (|r| > 0.70). Collinearity diagnostics showed a minimum tolerance of 0.530 and a maximum VIF of 1.888, indicating no evidence of problematic collinearity.

Independent variables significantly associated with metatarsal pain included the following:

- Isolated joint limitation (OR = 0.114, 95% CI: 0.030–0.442, *p* = 0.002), suggesting a strong negative association.
- MTP synovitis (2nd–5th joints) (OR = 2.093, 95% CI: 1.337–3.275, *p* = 0.001), indicating a higher likelihood of ARm.
- Severe hallux valgus (grades III–IV): grade III (OR = 10.026, 95% CI: 1.280–78.553, *p* = 0.028) and grade IV (OR = 5.428, 95% CI: 1.528–19.287, *p* = 0.009).
- Number of joints with ≥50% dislocation or subluxation (OR = 2.092, 95% CI: 1.275–3.432, *p* = 0.003).

Other variables, such as BMI, age, erosions (SENS scale), lesser toe deformities, and ankle or hindfoot involvement (e.g., Taylor’s bunion, rearfoot joint changes), were not significantly associated in the multivariate analysis.

A complete overview of regression coefficients, odds ratios, confidence intervals, and significance values is provided in Table 3.

## 4. Discussion

Despite achieving clinical remission, many patients with RA continue to experience foot pain [14,24,42], which can significantly impact their overall health perception. Our study specifically focused on patients with RA in remission who reported persistent foot pain, revealing that forefoot pain was the most prevalent, affecting 51% of the sample. This is consistent with previous studies that have also identified the forefoot as the most commonly affected region in RA when assessing the distribution of foot pain and damage [3,20,24,29,43].

Regarding the factors associated with foot pain, some studies consider it a global entity without distinguishing between anatomical regions, identifying variables such as BMI or general factors as contributors [19,24,44,45]. However, we believe that incorporating biomechanical and morphofunctional variables while specifying the exact location of pain can provide greater precision in identifying associated factors [46,47] and guide more targeted therapeutic strategies [48,49,50].

To the best of our knowledge, this is the first study to compare clinical, radiological, and biomechanical characteristics across different foot regions in patients with RA in remission, using a multivariate analysis. Previous studies have described the overall involvement of the foot [23,51] or have focused exclusively on the forefoot without exploring interregional comparisons [14,42,52]. Moreover, most of these studies have not incorporated biomechanical variables or multivariate analyses capable of identifying independent predictors according to anatomical regions. Understanding region-specific mechanisms can enhance and better guide both preventive strategies and treatments for patients with persistent foot pain.

Patients with metatarsal pain more frequently presented with moderate to severe hallux valgus, a greater number of joints with synovitis, and subluxations or dislocations exceeding 50%. These variables were independently associated with pain in the binary logistic regression analysis, suggesting a distinct structural and biomechanical pattern of forefoot pain in patients with RA. These findings are consistent with those of Matsumoto et al. [26], who described patterns of joint damage across foot regions using cluster analysis, highlighting the instability of the MTP joints and the central role of hallux valgus. Unlike that study, the present work incorporates pain as the primary variable of interest, providing a more clinically focused perspective on symptomatology. This highlights the importance of evaluating the presence and severity of hallux valgus, as well as the instability of the MTP joints in this specific pain location.

Interestingly, we also observed synovitis in patients without metatarsal pain, reinforcing the concept of subclinical inflammation during clinical remission [14,15,53,54]. This finding suggests that clinical examination should be complemented with ultrasound imaging to detect early signs of inflammatory activity, which could partly reflect the underlying immunopathology of RA [55]. However, our data also show that a higher number of joints with synovitis is associated with a painful pattern, further supporting the value of integrating ultrasound assessment [56] with targeted biomechanical evaluation.

Other variables, such as ankle dorsiflexion limitation, first metatarsophalangeal joint mobility, and subluxations of less than 50%, did not show significant differences between groups. Possible explanations for this lack of association include factors not assessed, such as the type of footwear used by the evaluated patients [50]; the high prevalence of plantar fasciitis in the control group of our sample, which has been associated with limited ankle dorsiflexion [57]; and the presence of substantial subtalar joint limitation, which may indicate a bony rather than muscular restriction in these patients.

Erosions were not significant in the logistic regression model but did appear in the bivariate comparison between groups. This may be partly explained by the moderate collinearity observed with dislocations/subluxations >50% (r = 0.580; *p* < 0.001), although the values did not reach thresholds typically considered problematic (|r| > 0.70 or VIF > 5). In fact, studies such as that of Siddle et al. [58] have demonstrated an association between plantar plate damage and the presence of erosions.

A limitation of this study is its cross-sectional design, which does not allow for the establishment of causal relationships. Additionally, the exclusive inclusion of patients with foot pain may have reduced the ability of some variables common to different types of pain to discriminate the specific location of pain. Some variables may be common to different types of foot pain; however, our comparison between patients with and without metatarsal pain (all of them with foot pain) allows for the identification of those factors particularly associated with metatarsal pain compared with pain in other locations. Footwear type and walking aid use were not recorded, which may have limited the assessment of their impact, despite excluding patients using orthoses or other orthopedic treatments within the previous three months. While this limitation does not compromise the validity of our conclusions, it highlights an area to be addressed in future research.

In summary, our model demonstrated that in RA patients in remission who experience foot pain, the presence of hallux valgus, synovitis, and dislocations exceeding 50% are significantly associated with forefoot pain. This suggests a distinct structural and biomechanical pattern that should be considered even during clinical remission. Unlike previous studies, such as Matsumoto et al. [26], who classified radiological damage patterns, our study incorporates pain as a central clinical variable, allowing for the identification of functional and structural predictors relevant to clinical practice. These findings emphasize the importance of integrating biomechanical, clinical, and ultrasound assessments in the evaluation of the rheumatoid foot, aiming to individualize therapeutic strategies and prevent the progression of painful alterations.

## 5. Conclusions

The findings of this study suggest that metatarsal pain in patients with RA in remission is not solely the result of accumulated structural damage but rather reflects a complex interaction between mechanical factors and residual inflammatory processes.

Moderate to severe hallux valgus, persistent synovitis, and severe dislocations should be considered specific therapeutic targets in the management of these patients, employing personalized podiatric interventions and treatment strategies tailored to individual characteristics.

The detection of subclinical inflammation in the foot using ultrasound as a complementary tool could enhance clinical control in RA patients in remission by identifying residual synovitis that may not be clinically apparent.

It would be advisable to adopt a multidisciplinary approach to the management of RA patients in remission, integrating specific clinical and biomechanical assessments of the foot to detect relevant deformities that may be associated with persistent pain.

Additional longitudinal studies are warranted to assess the progression of biomechanical alterations and their impact on pain development, providing a better understanding of the underlying pathogenic mechanisms and facilitating the development of more effective therapeutic interventions.

## Figures and Tables

**Table 1:** Pharmacological treatment and lifestyle habits by pain localization.

Variable	Overall (*n* = 118)	ARm (*n* = 61)	ARo (*n* = 57)	χ^2^ *p*-Value
	*n* (%)	*n* (%)	*n* (%)	
Corticosteroids	54 (45.8%)	27 (44.3%)	27 (47.4%)	0.735
Conventional DMARDs	69 (58.5%)	42 (68.9%)	27 (47.4%)	0.018
Biologic therapies	43 (36.4%)	20 (32.8%)	23 (40.4%)	0.394
NSAIDs	50 (42.3%)	24 (39.3%)	26 (45%)	0.491
Paracetamol	27 (22.9%)	8 (13.1%)	19 (33.3%)	0.009
Vitamin D supplements	42 (35.6%)	23 (37.7%)	19 (33.3%)	0.645
Calcium supplements	30 (25.4%)	14 (23.0%)	16 (28.1%)	0.670
Antiplatelet agents	2 (1.7%)	1 (1.6%)	1 (1.8%)	0.961
Hypothyroidism	15 (12.7%)	11 (18.0%)	4 (7.0%)	0.073
Hypertension	35 (29.7%)	19 (31.1%)	16 (28.1%)	0.715
Smoking	27 (22.9%)	16 (26.2%)	11 (19.3%)	0.510
Alcohol consumption	6 (5.1%)	3 (4.9%)	3 (5.3%)	0.996
Sedentary behavior	52 (44.1%)	27 (44.3%)	25 (43.9%)	0.865

Distribution of pharmacological treatment and lifestyle factors in rheumatoid arthritis (RA) patients with isolated metatarsal pain (ARm) versus those with pain in other foot regions (ARo). Values are expressed as *n* (%) per group. Abbreviations: ARm = RA with metatarsal pain; ARo = RA with other foot pain; DMARDs = disease-modifying antirheumatic drugs; NSAIDs = non-steroidal anti-inflammatory drugs.

**Table 2:** Clinical, biomechanical, and radiographic characteristics of the study groups.

	Overall (*n* = 118)	ARm (*n* = 61)	ARo (*n* = 57)	χ^2^/t-Test (*p*-Value)	95% CI (Lower–Upper)
	*n* (%)	*n* (%)	*n* (%)		
Manchester scale HAV				0.01 †	
HAV grade I:	71 (60.2)	29 (47.5%)	42 (73.7%)		
HAV grade II:	15 (12.7)	11 (18%)	4 (7%)		
HAV grades III and IV:	32 (27.1)	21 (34.4%)	11 (19.3%)		
Limited 1st MTP mobility	78 (66.1)	36 (59%)	42 (73.7%)	0.09 †	
Limited ankle dorsiflexion	87 (73.7)	44 (72.1%)	43 (75.4%)	0.68 †	
Lesser toe deformities	68 (57.6)	37 (60.7%)	31 (54.4%)	0.49 †	
Tailor’s bunion	38 (32.2)	19 (31.1%)	19 (33.3%)	0.80 †	
Type of foot					
Neutral	36 (30.5)	21 (34.4%)	15 (26.3%)	0.26 †	
Supinated	58 (49.2)	31 (50.8%)	27 (47.4%)		
Pronated	24 (20.3)	9 (14.8%)	15 (26.3%)		
STJ limitation	50 (42.4)	21 (34.4%)	29 (50.9%)	0.07 †	
		Media (SD)	Media (SD)	*p*-value	
Lesser toes deformities	2.21 (1.81)	2.27 (1.79)	2.14 (1.84)	0.341 *	[−0.527 to 0.803]
Erosions SENS scale	2.34 (1.76)	2.75 (1.80)	1.91 (1.62)	0.005 *	[0.214 to 1.470]
Subluxations (2nd–5th MTP, <50%)	0.68 (0.95)	0.60 (0.80)	0.77 (1.10)	0.175 *	[−0.515 to 0.184]
Dislocation (2nd–5th MTP, >50%)	0.39 (0.83)	0.55 (0.59)	0.29 (0.65)	0.013 *	[0.032 to 0.486]
Synovitis (2nd–5th MTP)	1.53 (1.41)	2.01 (1.38)	1.01 (1.27)	0.000 *	[0.513 to 1.485]

Notes: Comparison of clinical, biomechanical, and radiographic variables between RA patients with metatarsal pain (ARm) and those with pain in other foot regions (ARo). Results are expressed as *n* (%) or mean ± standard deviation. Statistical tests: † Chi-square test; * independent-samples t-test. Abbreviations: MTP = metatarsophalangeal joint; STJ = subtalar joint; HAV = hallux abductus valgus; SENS = Simple Erosion Narrowing Score; CI = confidence interval; SD = standard deviation.

**Table 3:** Binary logistic regression model for predictors of metatarsal pain.

	B	SE	*p* Value	OR	95% CI Para EXP (B)	
					Inferior	Superior
BMI	−0.133	0.074	0.075	0.876	0.757	1.013
Age	−0.049	0.031	0.110	0.952	0.897	1.011
Erosions (SENS)	0.138	0.183	0.452	1.148	0.802	1.642
STJ joint limitation	−2.169	0.690	0.002	0.114	0.030	0.442
MTP synovitis (2nd–5th)	0.738	0.229	0.001	2.093	1.337	3.275
Tailor’s bunion	−1.008	0.657	0.125	0.365	0.101	1.323
Ankle joint limitation	0.721	0.619	0.244	2.057	0.612	6.920
1st MTP limitation	−0.730	0.642	0.255	0.482	0.137	1.695
Type of foot (neutral)			0.764			
Pronated	−0.414	0.763	0.587	0.661	0.148	2.948
Supinated	0.120	0.648	0.853	1.128	0.317	4.014
Manchester scale HAV			0.013			
Grade II HAV	2.305	1.050	0.028	10.026	1.280	78.553
Grade III and IV HAV	1.692	0.647	0.009	5.428	1.528	19.287
MTP dislocations (2nd–5th)	0.738	0.253	0.003	2.092	1.275	3.432
Constant	4.897	2.765	0.077	133.914		

Multivariate binary logistic regression model identifying variables independently associated with metatarsal pain in RA patients. Odds ratios (OR), 95% confidence intervals (CI), and significance levels are shown for each variable. Abbreviations: B = regression coefficient; SE = standard error; BMI = Body Mass Index; SENS = Simple Erosion Narrowing Score; STJ = subtalar joint; MTP = metatarsophalangeal joint; 1st MTP = first metatarsophalangeal joint; HAV = hallux abductus valgus. Reference category for categorical variables: Type of foot—neutral; Manchester scale HAV—grade I.

## Data Availability

The datasets generated and analyzed during the current study are not publicly available due to patient confidentiality and institutional data protection policies but are available from the corresponding author on reasonable request.

## Abbreviations

The following abbreviations are used in this manuscript:

RA	Rheumatoid Arthritis
DAS28	Disease Activity Score-28
CDAI	Clinical Disease Activity Index
VAS	Visual Analog Scale
MTP	Metatarsophalangeal
STJ	Subtalar Joint
HAV	Hallux Abductus Valgus
Ankle	Ankle Joint
SD	Standard Deviation
OR	Odds Ratio
CI	Confidence Interval
SENS	Simple Erosion Narrowing Score
ARm	Rheumatoid Arthritis with metatarsal pain
ARo	Rheumatoid Arthritis with other foot pain
WHO	World Health Organization
EULAR	European Alliance of Associations for Rheumatology
OMERACT	Outcome Measures in Rheumatology
DMARDs	Disease-Modifying Antirheumatic Drugs

## References

[1] Black R.J., Cross M., Haile L.M., Culbreth G.T., Steinmetz J.D., Hagins H., A Kopec J., Brooks P.M., Woolf A.D., Ong K.L. (2023). Global, regional, and national burden of rheumatoid arthritis, 1990–2020, and projections to 2050: A systematic analysis of the Global Burden of Disease Study 2021. Lancet Rheumatol..

[2] GBD 2019 Diseases and Injuries Collaborators (2020). Global burden of 369 diseases and injuries in 204 countries and territories, 1990–2019: A systematic analysis for the Global Burden of Disease Study 2019. Lancet.

[3] Minaker K., Little H. (1973). Painful feet in rheumatoid arthritis. Can. Med. Assoc. J..

[4] Michelson J., Easley M., Wigley F.M., Hellmann D. (1994). Foot and Ankle Problems in Rheumatoid Arthritis. Foot Ankle Int..

[5] Bal A., Aydog E., Aydog S.T., Cakci A. (2006). Foot deformities in rheumatoid arthritis and relevance of foot function index. Clin. Rheumatol..

[6] Rome K., Gow P.J., Dalbeth N., Chapman J.M. (2009). Clinical audit of foot problems in patients with rheumatoid arthritis treated at Counties Manukau District Health Board, Auckland, New Zealand. J. Foot Ankle Res..

[7] Jacoby R.K., Jayson M.I.V., Cosh J.A. (1973). Onset, early stages, and prognosis of rheumatoid arthritis: A clinical study of 100 patients with 11-year follow-up. Br. Med. J..

[8] Fleming A., Crown J.M., Corbett M. (1976). Early rheumatoid disease. I. Onset. Ann. Rheum. Dis..

[9] Yano K., Ikari K., Inoue E., Sakuma Y., Mochizuki T., Koenuma N., Tobimatsu H., Tanaka E., Taniguchi A., Okazaki K. (2018). Features of patients with rheumatoid arthritis whose debut joint is a foot or ankle joint: A 5479-case study from the IORRA cohort. PLoS ONE.

[10] Lee S.W., Kim S.Y., Chang S.H. (2019). Prevalence of feet and ankle arthritis and their impact on clinical indices in patients with rheumatoid arthritis: A cross-sectional study. BMC Musculoskelet. Disord..

[11] Wechalekar M.D., Lester S., Proudman S.M., Cleland L.G., Whittle S.L., Rischmueller M., Hill C.L. (2012). Active foot synovitis in patients with rheumatoid arthritis: Applying clinical criteria for disease activity and remission may result in underestimation of foot joint involvement. Arthritis Rheum..

[12] Raffeiner B., Grisan E., Botsios C., Stramare R., Rizzo G., Bernardi L., Punzi L., Ometto F., Doria A. (2017). Grade and location of power Doppler are predictive of damage progression in rheumatoid arthritis patients in clinical remission by anti-tumour necrosis factor α. Rheumatology.

[13] Li Y., Zhang Z., Liu C., Kang Z., Li Z. (2024). Significance of High-Frequency Sonography for the Subclinical Progression of Rheumatoid Arthritis. J. Ultrasound Med. Off. J. Am. Inst. Ultrasound Med..

[14] Simonsen M.B., Hørslev-Petersen K., Cöster M.C., Jensen C., Bremander A. (2021). Foot and Ankle Problems in Patients with Rheumatoid Arthritis in 2019: Still an Important Issue. ACR Open Rheumatol..

[15] Ciurtin C., Jones A., Brown G., Sin F.E., Raine C., Manson J., Giles I. (2019). Real benefits of ultrasound evaluation of hand and foot synovitis for better characterisation of the disease activity in rheumatoid arthritis. Eur. Radiol..

[16] Woodburn J., Helliwell P.S. (1996). Relation between heel position and the distribution of forefoot plantar pressures and skin callosities in rheumatoid arthritis. Ann. Rheum. Dis..

[17] Louwerens J.W.K., Schrier J.C.M. (2013). Rheumatoid forefoot deformity: Pathophysiology, evaluation and operative treatment options. Int. Orthop..

[18] Kay J., Upchurch K.S. (2012). ACR/EULAR 2010 rheumatoid arthritis classification criteria. Rheumatology.

[19] Svensson B., Forslind K., Andersson M. (2020). Unacceptable pain in the BARFOT inception cohort of patients with rheumatoid arthritis: A long-term study. Scand. J. Rheumatol..

[20] Bouysset M., Bonvoisin B., Lejeune E., Bouvier M. (1987). Flattening of the Rheumatoid Foot in Tarsal Arthritis on X-ray. Scand. J. Rheumatol..

[21] Kerry R.M., Holt G.M., Stockley I. (1994). The foot in chronic rheumatoid arthritis: A continuing problem. Foot.

[22] Borman P., Ayhan F., Tuncay F., Sahin M. (2012). Foot Problems in a Group of Patients with Rheumatoid Arthritis: An Unmet Need for Foot Care. Open Rheumatol. J..

[23] Otter S.J., Lucas K., Springett K., Moore A., Davies K., Cheek L., Young A., Walker-Bone K. (2010). Foot pain in rheumatoid arthritis prevalence, risk factors and management: An epidemiological study. Clin. Rheumatol..

[24] González-Fernández M.L., Valor L., Morales-Lozano R., Hernández-Flórez D., López-Longo F.J., Martínez D., González C.M., Monteagudo I., Martínez-Barrio J., Garrido J. (2016). To what extent is foot pain related to biomechanical changes and ultrasound-detected abnormalities in rheumatoid arthritis?. Clin. Exp. Rheumatol..

[25] Stolt M., Kilkki M., Katajisto J., Suhonen R. (2021). Self-assessed foot health in older people with rheumatoid arthritis-A cross-sectional study. Int. J. Older People Nurs..

[26] Matsumoto T., Nakamura I., Miura A., Momoyama G., Ito K. (2014). Radiologic patterning of joint damage to the foot in rheumatoid arthritis. Arthritis Care Res..

[27] Índice de la Actividad de la Enfermedad de Artritis Reumatoide DAS-28. https://www.msdmanuals.com/medical-calculators/RheumatoidArthritisDAS28-es.htm.

[28] Pincus T., Yazici Y., Bergman M.J. (2009). RAPID3, an Index to Assess and Monitor Patients with Rheumatoid Arthritis, Without Formal Joint Counts: Similar Results to DAS28 and CDAI in Clinical Trials and Clinical Care. Rheum. Dis. Clin. N. Am..

[29] Vidigal E., Jacoby R.K., Dixon S.A.J., Ratliff A.H., Kirkup J. (1975). The foot in chronic rheumatoid arthritis. Ann. Rheum. Dis..

[30] WHO (2020). WHO Guidelines on Physical Activity and Sedentary Behaviour: Web Annex Evidence Profiles.

[31] José Cid C., Juan Pablo Acuña B., Javier de Andrés A., Luis Díaz J., Leticia Gómez-Caro A. (2014). ¿Qué y cómo evaluar al paciente con dolor crónico? evaluación del paciente con dolor crónico. Rev. Médica Clínica Las Condes.

[32] Pasarín Martínez A., Álvarez Goenaga F., Ruiz Nasarre A., Fernández N.A., Sarró Maluquer S., Fierro D.C. (2020). Valoración de la fiabilidad intra-e interobservador de la medición de la retracción gemelar utilizando el test de Silfverskiöld y el test en posición neutra del pie. Rev. Pie Tobillo.

[33] Singh D. (2013). Nils Silfverskiöld (1888–1957) and gastrocnemius contracture. Foot Ankle Surg..

[34] Sánchez-Gómez R., Becerro-de-Bengoa-Vallejo R., Losa-Iglesias M.E., Calvo-Lobo C., Navarro-Flores E., Palomo-López P., Romero-Morales C., López-López D. (2020). Reliability Study of Diagnostic Tests for Functional Hallux Limitus. Foot Ankle Int..

[35] Coughlin M.J., Shurnas P.S. (2004). Hallux rigidus. J. Bone Jt. Surg..

[36] Del Vecchio J.J., Dalmau-Pastor M. (2024). Anatomy, Biomechanics, and Pathogenesis of the Lesser Toes Deformities. Foot Ankle Clin..

[37] Ajis A., Koti M., Maffulli N. (2005). Tailor’s Bunion: A Review. J. Foot Ankle Surg..

[38] Garrow A.P., Papageorgiou A., Silman A.J., Thomas E., Jayson M.I.V., Macfarlane G.J. (2001). The grading of hallux valgus. The Manchester Scale. J. Am. Podiatr. Med. Assoc..

[39] D’Agostino M.A., Terslev L., Aegerter P., Backhaus M., Balint P., Bruyn G.A., Filippucci E., Grassi W., Iagnocco A., Jousse-Joulin S. (2017). Scoring ultrasound synovitis in rheumatoid arthritis: A EULAR-OMERACT ultrasound taskforce—Part 1: Definition and development of a standardised, consensus-based scoring system. RMD Open.

[40] Vargas Guerrero A., Pineda Villaseñor C. (2006). Evaluación radiográfica del daño anatómico en la artritis reumatoide. Rev. Colomb. Reumatol..

[41] Ohashi H., Nishida K., Nasu Y., Saiga K., Nakahara R., Horita M., Okita S., Ozaki T. (2021). A Novel Radiographic Measurement Method for the Evaluation of Metatarsophalangeal Joint Dislocation of the Lesser Toe in Patients with Rheumatoid Arthritis. Int. J. Environ. Res. Public Health.

[42] van der Leeden M., Steultjens M.P.M., van Schaardenburg D., Dekker J. (2010). Forefoot disease activity in rheumatoid arthritis patients in remission: Results of a cohort study. Arthritis Res. Ther..

[43] Stolt M., Suhonen R., Leino-Kilpi H. (2017). Foot health in patients with rheumatoid arthritis—A scoping review. Rheumatol. Int..

[44] Kim H.A., Park S.Y., Shin K. (2022). Implications of Persistent Pain in Patients with Rheumatoid Arthritis Despite Remission Status: Data From the KOBIO Registry. J. Rheum. Dis..

[45] Dahmen R., Konings-Pijnappels A., Kerkhof S., Verberne S., Boers M., Roorda L.D., van der Leeden M. (2020). Higher body mass index is associated with lower foot health in patients with rheumatoid arthritis: Baseline results of the Amsterdam-Foot cohort. Scand. J. Rheumatol..

[46] Sánchez-Rodríguez R., Martínez-Quintana R., Martínez-Nova A., Martínez-Rico M., Pedrera-Zamorano J.D., Chicharro-Luna E. (2023). Correlation between the foot pressure index and the prevalence of plantar hyperkeratosis. J. Tissue Viability.

[47] Barn R., Turner D.E., Rafferty D., Sturrock R.D., Woodburn J. (2013). Tibialis posterior tenosynovitis and associated pes plano valgus in rheumatoid arthritis: Electromyography, multisegment foot kinematics, and ultrasound features. Arthritis Care Res..

[48] Macarrón Pérez P., Morales Lozano M.d.R., Vadillo Font C., Abásolo Alcázar L., Martínez Rincón C., Fernández Gutiérrez B., Blanco Hontiyuelo M., González-Fernández M.L. (2021). Multidisciplinary approach in the treatment of tendinous foot involvement in rheumatoid arthritis. Clin. Rheumatol..

[49] Bartolo D., Galea A.M., Formosa C., Gatt A. (2022). The Management of Metatarsalgia in Rheumatoid Arthritis Using Simple Insoles: An Effective Concurrent Treatment to Drug Therapy. J. Am. Podiatr. Med. Assoc..

[50] Cho N.S., Hwang J.H., Chang H.J., Koh E.M., Park H.S. (2009). Randomized controlled trial for clinical effects of varying types of insoles combined with specialized shoes in patients with rheumatoid arthritis of the foot. Clin. Rehabil..

[51] Grondal L., Tengstrand B., Nordmark B., Wretenberg P., Stark A. (2008). The foot: Still the most important reason for walking incapacity in rheumatoid arthritis: Distribution of symptomatic joints in 1000 RA patients. Acta Orthop..

[52] Turner D.E., Helliwell P.S., Emery P., Woodburn J. (2006). The impact of rheumatoid arthritis on foot function in the early stages of disease: A clinical case series. BMC Musculoskelet. Disord..

[53] Nguyen H., Ruyssen-Witrand A., Gandjbakhch F., Constantin A., Foltz V., Cantagrel A. (2014). Prevalence of ultrasound-detected residual synovitis and risk of relapse and structural progression in rheumatoid arthritis patients in clinical remission: A systematic review and meta-analysis. Rheumatology.

[54] Wechalekar M.D., Lester S., Hill C.L., Lee A., Rischmueller M., Smith M.D., Walker J.G., Proudman S.M. (2016). Active Foot Synovitis in Patients With Rheumatoid Arthritis: Unstable Remission Status, Radiographic Progression, and Worse Functional Outcomes in Patients With Foot Synovitis in Apparent Remission. Arthritis Care Res..

[55] Orange D.E., Agius P., Dicarlo E.F., Mirza S.Z., Pannellini T., Ma J.S., Jiang C.S., Figgie M.P., Frank M.O., Robinson W.H. (2019). Histologic and transcriptional evidence of sub-clinical synovial inflammation in patients with rheumatoid arthritis in clinical remission. Arthritis Rheumatol..

[56] Son K.M., Song S.H., Lim S.K., Seo Y.I., Kim H.A. (2012). Characteristics of patients with rheumatoid arthritis in clinical remission: The many aspects of DAS28 remission. Clin. Exp. Rheumatol..

[57] García Vidal J.-A., Piñero Palazón J.-G., Baño Alcaraz A., Sánchez Martínez M.-P., Medina i Mirapeix F. (2019). Valor del Test de Silfverskiöld para el diagnóstico de la fascitis plantar. Rev. Int. Cienc. Podol..

[58] Siddle H.J., Hodgson R.J., Hensor E.M.A., Grainger A.J., Redmond A.C., Wakefield R.J., Helliwell P.S. (2017). Plantar plate pathology is associated with erosive disease in the painful forefoot of patients with rheumatoid arthritis. BMC Musculoskelet. Disord..

